# Taxonomy and SSU rDNA-Based Phylogeny of Two Heterotrich Ciliates (Ciliophora, Heterotrichea) Collected From Subtropical Wetlands of China, Including the Description of a New Species, *Linostomella pseudovorticella* n. sp.

**DOI:** 10.3389/fmicb.2021.719360

**Published:** 2021-09-07

**Authors:** Didi Jin, Xuetong Zhao, Tingting Ye, Jie Huang, Alan Warren, Saleh A. Al-Farraj, Xiangrui Chen

**Affiliations:** ^1^School of Marine Sciences, Ningbo University, Ningbo, China; ^2^Laboratory of Protozoological Biodiversity and Evolution in Wetland, College of Life Sciences, Shaanxi Normal University, Xi’an, China; ^3^Key Laboratory of Aquatic Biodiversity and Conservation of Chinese Academy of Sciences, Institute of Hydrobiology, Chinese Academy of Sciences, Wuhan, China; ^4^Department of Life Sciences, Natural History Museum, London, United Kingdom; ^5^Zoology Department, College of Science, King Saud University, Riyadh, Saudi Arabia

**Keywords:** molecular phylogeny, morphology, new species, subtropical wetland, ciliate

## Abstract

The Heterotrichea Stein, 1859 are a group of ciliated protists (single-celled eukaryotes) that occur in a wide variety of aquatic habitat where they play important roles in the flow of nutrients and energy within the microbial food web. Many species are model organisms for research in cytology and regenerative biology. In the present study, the morphology and phylogeny of two heterotrich ciliates, namely, *Linostomella pseudovorticella* n. sp. and *Peritromus kahli* Villeneuve-Brachon, 1940, collected from subtropical wetlands of China, were investigated using morphological and molecular methods. *L. pseudovorticella* n. sp. differs from its only known congener, *Linostomella vorticella* Ehrenberg, 1833 Aescht in Foissner et al., 1999, by having more ciliary rows (48–67, mean about 56 vs. 26–51, mean about 42) and its small-subunit (SSU) rDNA sequence, which shows a 15-bp divergence. Although *P. kahli* has been reported several times in recent decades, its infraciliature has yet to be described. A redescription and improved diagnosis of this species based on a combination of previous and present data are here supplied. Phylogenetic analyses based on SSU rDNA sequences revealed that the genus *Linostomella* is positioned within Condylostomatidae, and Peritromidae is sister to Climacostomidae with relatively low support, and the family Spirostomidae is the root branch of the class Heterotrichea.

## Introduction

Ciliated protists (ciliates) are a morphologically diverse and highly specialized group of microbial eukaryotes that constitute an important component of the microbial food web (Azam et al., 1983; Dolan et al., 2012; Miao et al., 2020; Zhao et al., 2020; Liu et al., 2021). Members of the ciliate class Heterotrichea Stein, 1859 are characterized by their typically large body, prominent oral apparatus, and somatic kineties composed of dikinetids with postciliodesmata (Lynn, 2008; Song et al., 2009; Shazib et al., 2014; Hu et al., 2019). The Heterotrichea contains 10 families and about 60 genera, several of which are well-known, e.g., *Stentor* Oken, 1815 and *Spirostomum* Ehrenberg, 1834. In contrast, some genera are not familiar because they have few species and/or they are difficult to collect, e.g., *Linostomella* Aescht in Foissner et al., 1999, *Chattonidium* Villeneuve, 1937, and *Peritromus* Stein, 1863 (Rosati et al., 2004; Modeo et al., 2006; Chi et al., 2020, 2021).

The genus *Linostomella* is monotypic and classified within the family Condylostomatidae Kahl in Doflein and Reichenow, 1929. The type species, *Linostomella vorticella* Ehrenberg, 1833 Aescht in Foissner et al., 1999, has been recorded and redescribed several times since it was first reported by Ehrenberg (Ehrenberg, 1833; Dujardin, 1841; Jankowski, 1978; Aescht, 2001; Rossi et al., 2016). Recently, Chi et al. (2020) gave a detailed redescription of *L. vorticella* and investigated its molecular phylogeny based on a population from Qingdao, China.

The genus *Peritromus* is characterized by the strongly dorso-ventrally flattened body and the ciliary pattern on dorsal and ventral sides being obviously differentiated (Song and Wilbert, 1997). Although 16 nominal species of *Peritromus* have been reported, detailed morphological information and molecular data are available for only two, i.e., *Peritromus faurei* Kahl, 1932 and *Peritromus kahli* Villeneuve-Brachon, 1940 (Song and Wilbert, 1997; Rosati et al., 2004; Miao et al., 2009). It has been hypothesized that Peritromidae (*Peritromus*) is the ancestral taxon of Heterotrichea (Chi et al., 2021), although this finding is inconsistent with most other molecular phylogenetic trees (Yan et al., 2015; Fernandes et al., 2016; Chen et al., 2017). Therefore, the phylogenetic position of Peritromidae remains ambiguous.

**FIGURE 1:**
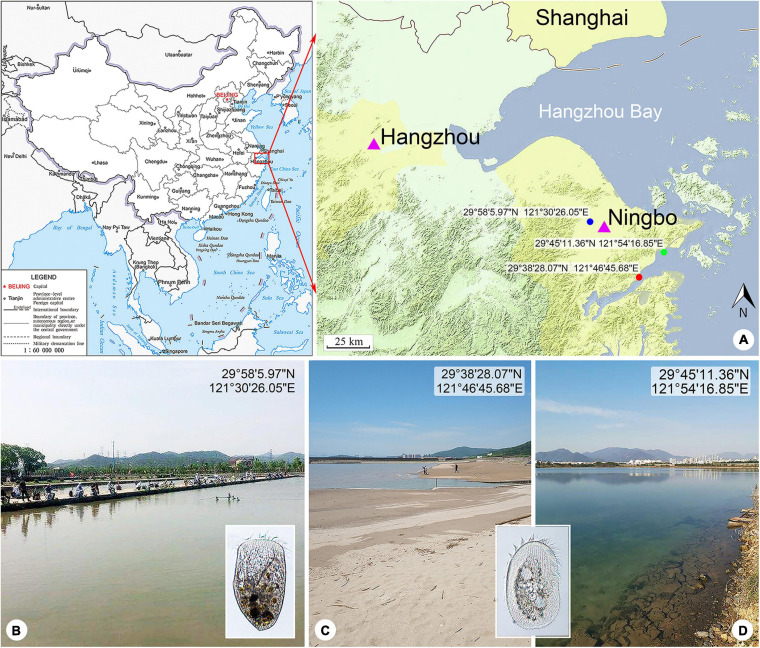
Locations of the sample sites. **(A)** Maps showing the locations of the freshwater pond (blue spot), coastal brackish lake (green spot), and Xiangshan Bay (red spot). The map of China [drawing review number: GS (2019)1652] cited from the MAP WORD (www.tianditu.gov.cn). **(B)** Freshwater pond near Yong River where *Linostomella pseudovorticella* n. sp. was collected. **(C)** Intertidal sandy beach near Xiangshan Bay where *Peritromus kahli* population-II was collected. **(D)** Brackish lake near Meishan Island where *Peritromus kahli* population-I was collected.

In the present study, two heterotricheans, *L. pseudovorticella* n. sp. and *P. kahli*, were isolated from subtropical wetlands in Ningbo, China (Figure 1). Their taxonomy and phylogeny were investigated based on detailed morphological information and small-subunit (SSU) rDNA sequences.

## Materials and Methods

### Sample Collection, Observation, and Identification

*Linostomella pseudovorticella* n. sp. was collected from a freshwater pond near Yong River (N29°58′5.97″; E121°30′26.05″), Ningbo, China on March 6, 2018. The water temperature was about 12.5°C. Samples were collected from the upper layer of water using a 20-μm mesh plankton net.

*Peritromus kahli* population-I was collected from a brackish lake near Meishan Island (N29°45′11.36″; E121°54′16.85″), Ningbo, China on May 20, 2020, when the water temperature was about 27.0°C and salinity was about 14.1 PSU. Population-II was collected from the intertidal zone of a sandy beach at Xiangshan Bay (N29°38′28.07″; E121°46′45.68″), Ningbo, China on May 21, 2019, when the water temperature was about 24.0°C and salinity was about 17.6 PSU. Samples were transferred to the laboratory with some pieces of aquatic plant stems and leaves collected from the same habitat.

The behavior of both species was studied in Petri dishes under a dissecting microscope. Their morphology *in vivo* was observed using bright field and differential interference contrast microscopy (Leica DM2500, Germany) at 100–1,000 × magnifications. The ciliary pattern and nuclear apparatus were revealed by protargol staining (Wilbert, 1975). Terminology followed Song and Wilbert (1997); Rosati et al. (2004), and Chi et al. (2020).

### DNA Extraction, Gene Amplification, and Sequencing

Clonal cultures of neither species could be established. Thus, single cells of each species were isolated from the original sample, washed three times with filtered habitat water (0.22-μm pore size membrane, Millipore, United States) and twice using ultrapure water, and placed in 1.5-ml microfuge tubes with a minimum volume of water. Genomic DNA was extracted using a DNeasy Blood and Tissue Kit (Qiagen, Hilden, Germany) according to the manufacturer’s instructions. The SSU rDNA was amplified with the universal eukaryotic primers 18SF (5′-AAC CTG GTT GAT CCT GCC AGT-3′) and 18SR (5′-TGA TCC TTC TGC AGG TTC ACC TAC-3′) (Medlin et al., 1988).

The polymerase chain reaction (PCR) conditions for the amplification of SSU rDNA sequences were as follows: a cycle of initial denaturation at 98°C for 30 s, followed by 35 cycles of amplification (98°C, 10 s; 56°C, 20 s; 72°C, 100 s), and a final extension at 72°C for 120 s. Q5 Hot Start High-Fidelity DNA Polymerase (NEB Co., Ltd., M0493, Beijing, China) was used to minimize the possibility of PCR amplification errors. PCR products were checked using agarose gel and were sequenced in TSINGKE (Hangzhou, China). Sequence fragments were assembled into contigs using Seqman (DNAStar).

### Phylogenetic Analyses

Phylogenetic analyses of SSU rDNA sequences were performed using an alignment comprising 69 representative sequences of Heterotrichea (Supplementary Table 1) and six sequences of Karyorelictea as the outgroup. All sequences were aligned with the MAFFT algorithm applying the default parameters provided on the GUIDANCE web server^1^ (Penn et al., 2010a, b). The ends of alignments were trimmed by BioEdit v.7.1.3.0 (Hall, 1999). Hypervariable sites were removed using Gblocks version 0.91b with default setting^2^ (Castresana, 2000; Talavera and Castresana, 2007), which resulted in a matrix of 1,552 characters.

Maximum likelihood (ML) analyses were conducted on CIPRES Science Gateway with RAxML-HPC2 on XSEDE v.8.2.11 (Stamatakis et al., 2008) using the GTR + I + G model as optimal choice according to the Akaike information criterion (AIC) criterion by ModelTest v.3.4 (Posada and Crandall, 1998). Support for the best ML tree was from 1,000 bootstrap replicates. A Bayesian inference (BI) analysis was performed on CIPRES Science Gateway^3^ with MrBayes on XSEDE v.3.2.7 (Ronquist and Huelsenbeck, 2003) using the GTR + I + G model (selected by MrModelTest v.2.3) (Nylander, 2004). The chain length of Markov chain Monte Carlo simulations was 10^6^ generations with a sampling frequency of every 100th generation. The first 25% of sampled trees were discarded as burn-in. Phylogenetic trees were visualized *via* MEGA v.5.0 (Tamura et al., 2011) and TreeView v.1.6.6 (Page, 1996). Systematic classification mainly followed Lynn (2008); Gao et al. (2016), and Fernandes et al. (2016).

## Results

### ZooBank Registration

Present work: urn:lsid:zoobank.org:pub:FDBA64CC-21E7-4484-A2B5-7DE4306BCAFF.

*Linostomella pseudovorticella* n. sp.: urn:lsid:zoobank. org:act:0FE6B19E-4FAD-46AD-AB05-364FEF7831AC.

Family Condylostomatidae Kahl in Doflein and Reichenow, 1929.

Genus *Linostomella* Aescht in Foissner et al., 1999.

*Linostomella pseudovorticella* n. sp.

### Diagnosis

Body 135–190 μm × 85–125 μm *in vivo*; ovoidal with a truncated anterior end and a rounded posterior end; conspicuous depression at posterior end; oral region large and deep; macronucleus moniliform with 6–15 interconnected nodules of similar size and shape; single subterminal contractile vacuole; cortical granules ellipsoidal, black-greenish in color; 42–59 adoral zone of membranelles; 48–67 somatic kineties; paroral membrane prominent and composed of two parallel rows of kineties; freshwater habitat.

### Type Locality

A subtropical freshwater pond near Yong River (N29°58′5.97″; E121°30′26.05″), Ningbo, China.

### Type Specimens

One protargol slide with the holotype specimen circled in ink (registration number: JDD-20180306-01) and two slides with protargol-stained paratype specimens (registration numbers: JDD-20180306-02, JDD-20180306-03) have been deposited in the Laboratory of Protozoology, Ocean University of China (OUC).

### Etymology

The species-group name *pseudovorticella* is a composite of the Greek adjective *pseudo*- (wrong, lying) and the species group name *vorticella*, referring to the similar morphology between *L. vorticella* and *L. pseudovorticella*.

### Morphological Description

**FIGURE 2:**
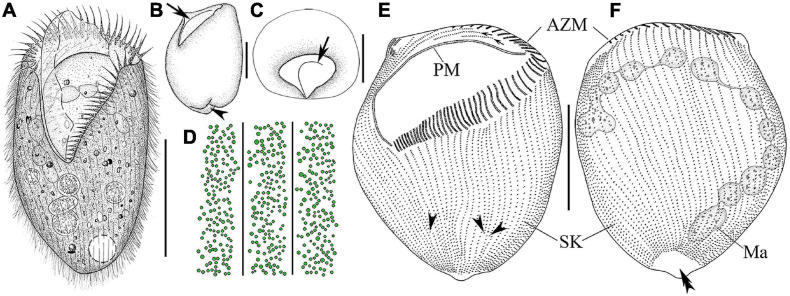
Morphology and infraciliature of *Linostomella pseudovorticella* n. sp. **(A–D)** *in vivo* and **(E**,**F)** after protargol staining. **(A)** Ventral view of a representative individual. **(B)** Left lateral view of a living individual; arrowhead indicates the depression at posterior end of body, and arrow indicates the paroral membrane. **(C)** Apical view of a living cell; arrow indicates the paroral membrane. **(D)** Distribution of cortical granules on ventral side. **(E**,**F)** Ventral **(E)** and dorsal **(F)** view of the infraciliature of the holotype specimen; arrowheads indicate the locations where shortened kineties terminate posteriorly, and double arrowhead indicates the glabrous area. Abbreviations: AZM, adoral zone of membranelles; Ma, macronucleus; PM, paroral membrane; SK, somatic kineties. Scale bars = 100 μm **(A**,**E**,**F)**, 50 μm **(B**,**C)**.

**FIGURE 3:**
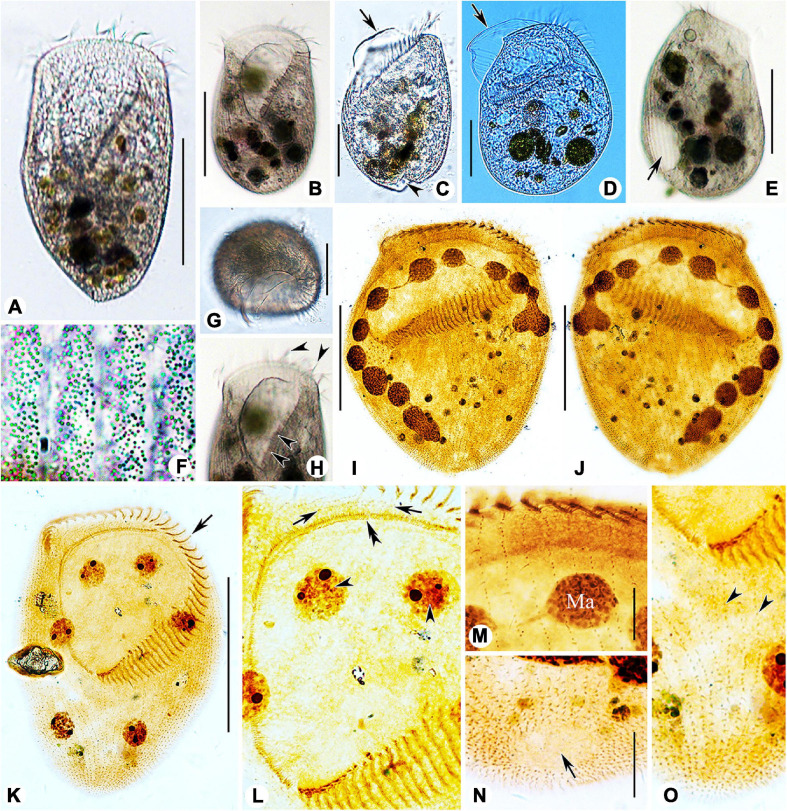
Photomicrographs of *Linostomella pseudovorticella* n. sp. **(A–H)** from life and **(I–O)** after protargol staining. **(A**,**B)** Ventral view of two typical individuals. **(C**,**D)** Left lateral view, showing the prominent paroral membrane (arrows) and the depression at posterior end of body (arrowhead). **(E)** Right lateral view, showing the contractile vacuole (arrow). **(F)** To show the arrangement of the small cortical granules. **(G)** Apical view of an individual. **(H)** Anterior region of cell, arrowheads indicate the adoral zone of membranelles. **(I**,**J)** Ventral **(I)** and dorsal **(J)** view of the infraciliature of the holotype specimen. **(K)** Ventral view of the infraciliature; arrow indicates the adoral zone of membranelles. **(L)** Detail of oral area; double arrowhead indicates the paroral membrane, arrows indicate the short kineties, and arrowheads indicate the macronuclear nodules. **(M)** Anterior portion of dorsal view of the cell. **(N)** Dorsal view showing glabrous area at posterior end of cell (arrow). **(O)** Ventral view of posterior portion of cell; arrowheads indicate the short somatic kineties. Abbreviation: Ma, macronucleus. Scale bars = 100 μm **(A**,**B**,**E**,**I–K)**, 50 μm **(C**,**D**,**G)**, and 20 μm **(M**,**N)**.

Cell size about 135–190 μm × 85–125 μm *in vivo* and 135–245 μm × 105–205 μm after protargol staining (Table 1). Body ovoid in outline, widest in mid-body region, anterior end truncated and with a V-shaped oral opening, posterior end narrowly rounded and with a conspicuous depression (Figures 2A,B, 3A–C). Oral region large and deep, extending from anterior end to mid-body region (Figures 2A,C, 3A,B,G,H). Pellicle thick, furnished with numerous spherical, black-greenish cortical granules, about 0.3–0.5 μm in diameter, densely distributed between ciliary rows (Figures 2D, 3F). Cytoplasm colorless, filled with numerous food particles and algae rendering cell slightly grayish when viewed at low magnifications (Figures 3B–E). Single large contractile vacuole, subterminally positioned (Figures 2A, 3E). Macronucleus moniliform, with 6–15 ellipsoidal nodules, usually arranged in a longitudinally oriented “C” shape (Figures 2F, 3J); occasionally interspersed throughout cell (in 5 out of 30 cells examined) (Figure 3K). Locomotion by swimming in upper layer of water while rotating about main body axis.

**TABLE 1:** Morphometric data on *Linostomella pseudovorticella* n. sp. (*L. pse*) and *Peritromus kahli* population-I, Meishan Island population (*P. kah-*I), and population-II, Xiangshan Bay population (*P. kah-*II).

Character	Species	Min	Max	Mean	M	SD	SE	CV	*n*
Body, length (μm)	*L. pse*	134	243	186.5	190.5	27.8	5.4	14.9	26
	*P. kah*-I	80	145	100.6	97.5	16.5	4.1	16.4	16
	*P. kah*-II	95	198	138.3	141.5	26.3	5.2	19.0	26
Body, width (μm)	*L. pse*	104	204	158.2	157.0	26.1	5.1	16.5	26
	*P. kah*-I	61	93	78.6	78.0	9.9	2.5	12.6	16
	*P. kah*-II	63	127	98.3	98.0	16.2	3.2	16.5	26
Oral area, length (μm)	*L. pse*	51	124	87.7	87.5	18.5	3.6	21.1	26
	*P. kah*-I	41	88	62.2	60.5	13.6	3.4	21.9	16
	*P. kah*-II	54	126	82.9	81.5	16.3	3.2	19.7	26
Adoral membranelles, number	*L. pse*	42	59	50.4	51.5	5.2	1.0	10.4	26
	*P. kah*-I	63	84	74.2	75.0	7.0	2.0	9.4	12
	*P. kah*-II	71	94	83.6	83.0	5.3	1.0	6.3	26
Somatic kineties, number	*L. pse* ^a^	48	67	56.5	56.0	5.7	1.1	10.0	26
	*P. kah*-I^b^	17	24	20.7	21.5	1.4	0.5	6.9	10
	*P. kah*-II^b^	21	28	24.0	24.0	1.9	0.4	8.1	26
Preoral kineties, number	*L. pse*	–	–	–	–	–	–	–	–
	*P. kah*-I	3	8	5.3	5.0	1.4	0.5	26.8	10
	*P. kah*-II	4	7	5.0	5.0	0.7	0.1	13.9	26
Postoral kineties, number	*L. pse*	–	–	–	–	–	–	–	–
	*P. kah*-I	6	17	10.4	10.0	3.5	1.1	33.7	10
	*P. kah*-II	11	22	14.9	14.5	2.9	0.6	19.7	26
Cilia in dorsal kinety, number	*L. pse*	–	–	–	–	–	–	–	–
	*P. kah*-I	17	25	21.0	20.5	3.5	1.4	16.8	6
	*P. kah*-II	17	34	23.6	23.0	4.0	1.0	16.8	17
Ma nodules, number	*L. pse*	6	15	9.3	9.0	2.1	0.4	22.8	26
	*P. kah*-I	2	2	2.0	2.0	0.0	0.0	0.0	16
	*P. kah*-II	2	2	2.0	2.0	0.0	0.0	0.0	26
Ma, length (μm)	*L. pse*	16	35	22.9	22.0	5.1	1.0	22.1	26
	*P. kah*-I	17	36	26.5	26.0	4.7	1.2	17.7	15
	*P. kah*-II	15	24	18.7	19.0	2.7	0.5	14.4	26
Ma, width (μm)	*L. pse*	12	31	19.2	22.0	5.1	1.0	26.3	26
	*P. kah*-I	15	28	21.2	21.0	3.7	1.0	17.6	15
	*P. kah*-II	13	19	15.4	15.0	1.8	0.4	11.8	26
Mi, number	*L. pse*	–	–	–	–	–	–	–	–
	*P. kah*-I	1	1	1.0	1.0	0.0	0.0	0.0	3
	*P. kah*-II	1	3	1.7	2.0	0.6	0.2	37.0	15
Mi, diameter (μm)	*L. pse*	–	–	–	–	–	–	–	–
	*P. kah*-I	6	7	6.7	7.0	0.6	0.3	8.7	3
	*P. kah*-II	3	7	4.3	4.0	1.2	0.3	28.5	15
Oral area fiber bundles, number	*L. pse*	–	–	–	–	–	–	–	–
	*P. kah*-I	18	23	20.0	19.0	2.7	1.5	13.2	3
	*P. kah*-II	16	23	19.8	20.0	2.4	0.5	12.3	24

*All data based on protargol-stained specimens.*

*CV, coefficient of variation in%; M, Median; Ma, macronucleus; Max, maximum; Mean, arithmetic mean; Mi, micronucleus; Min, minimum; n, number of specimens investigated; SD, standard deviation; SE, Standard Error.*

*^a^Includes bipolar and shortened somatic kineties.*

*^b^Refers only to the number of bipolar kineties—preoral kineties, postoral kineties, and caudal margin kinety were excluded.*

Infraciliature as shown in Figures 2E,F, 3I–O. Adoral zone composed of 42–59 membranelles (Table 1), with cilia 20–40 μm in length, commences subapically near right margin, terminates in mid-body region (Figures 2E,F, 3H–K). Paroral membrane easily recognized *in vivo* and very conspicuous in protargol-stained cells, with cilia 30–40 μm in length, located on right of adoral zone of membranelles, composed of two parallel rows of kinetosomes (Figures 2A,E, 3H,L). In total, 48–67 somatic kineties composed of dikinetids, only one kinetosome of each dikinetid bears a cilium that is about 8–10 μm long *in vivo* (Figures 2E,F). Two short kineties located between adoral zone of membranelles and paroral membrane (Figures 2E, 3L); several shortened somatic kineties near central axis on ventral side, not forming a suture (Figures 2E, 3K,O). Dorsal kineties bipolar, evenly spaced (Figures 2F, 3J,M,N). Glabrous area at posterior end of cell, about ∼30 μm × 10 μm after protargol staining (Figures 2F, 3N).

Family Peritromidae Stein, 1867.

Genus *Peritromus* Stein, 1863.

*Peritromus kahli* Villeneuve-Brachon, 1940.

*Peritromus kahli* had been reported several times but mostly in ecological studies or faunal surveys. Consequently, several morphological characters remain unknown. An improved diagnosis is here supplied based on the present and previous studies.

### Improved Diagnosis

Size 100–175 μm × 65–110 μm *in vivo*; body ovoidal with rounded ends; length to width ratio approximately 1.5:1; strongly dorsoventrally flattened with an irregular hump on dorsal side; two ellipsoidal macronuclear nodules; two types cortical granules, type I dark grayish, type II dark greenish; 63–94 adoral membranelles; paroral membrane single-rowed; 16–23 fiber bundles; 3–8 preoral kineties; 6–22 postoral kineties; 17–28 bipolar kineties and 1 caudal margin kinety; dorsal kineties composed of one external kinety and one internal kinety; marine habitat.

### Voucher Slides

Eight protargol slides (four slides for each population) have been deposited as voucher material in the Laboratory of Protozoology, OUC (population I registration number: ZXT-20200520-01, 02, 03, 04; population II YTT-20200250-01, 02, 03, 04).

### Morphological Description of Ningbo Population-I

**FIGURE 4:**
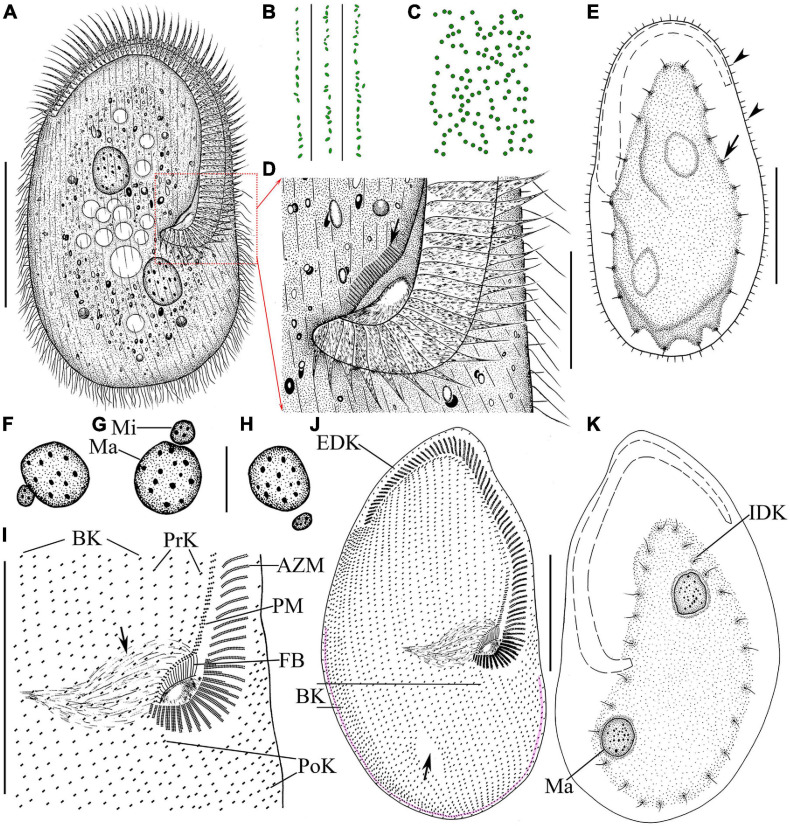
Morphology and infraciliature of *Peritromus kahli* population-I [**(A–E)**, *in vivo*] and population-II [**(F–K)**, after protargol staining]. **(A)** Ventral view of a typical individual. **(B,C)** The arrangement of the type I (smaller, oval) and type II (larger, round) cortical granules. **(D)** Detail of oral area; arrow indicates fiber bundles. **(E)** Dorsal view of an individual; arrowheads indicate the external dorsal kinetids, and arrow indicates the internal dorsal kinetids. **(F–H)** Various juxtapositions of macronuclear nodules and micronuclei. **(I)** Detail of oral area showing the infraciliature; arrow indicates cytopharynx. **(J**,**K)** Ventral **(J)** and dorsal **(K)** view of the infraciliature; the pink line depicts the caudal margin kinety, and arrow indicates a wide gap (putative cytoproct). Abbreviations: AZM, adoral zone of membranelles; BK, bipolar kineties; EDK, external dorsal kinety; FB, fiber bundles; IDK, internal dorsal kinety; Ma, macronucleus; Mi, micronucleus; PoK, postoral kineties; PM, paroral membrane; PrK, peroral kineties. Scale bars = 50 μm **(A**,**E**,**I–K)**, 15 μm **(D**,**F–H)**.

**FIGURE 5:**
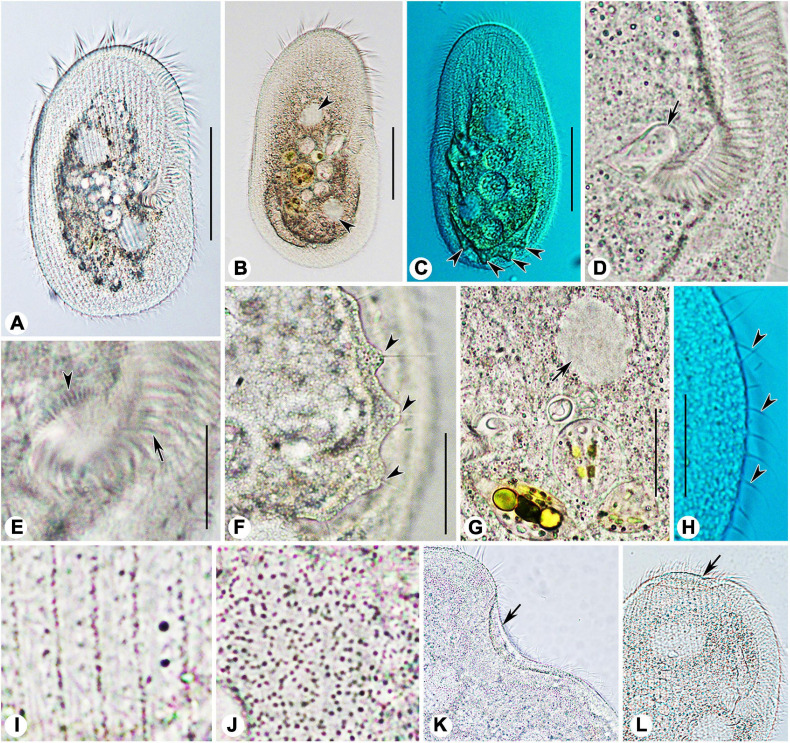
Photomicrographs of *Peritromus kahli* **(A**,**C–J)** population-I and **(B,K,L)** population-II from life. **(A**,**B)** Ventral view of two typical individuals; arrowheads indicate macronuclear nodules. **(C)** Dorsal view of an individual; arrowheads indicate wart-like prominences (papillae). **(D)** Detail of oral area; arrow indicates cytostome. **(E)** Enlargement of the oral area; arrow indicates adoral zone of membranelles, and arrowhead indicates fiber bundles. **(F)** Dorsal view of wart-like prominences (papillae) from which spine-like cilia (dorsal bristles) emerge (arrowheads). **(G)** To show a macronuclear nodule (arrow). **(H)** To show the cilia of external dorsal kinetids (arrowheads) distributed along the edge of the cell. **(I)** Ventral view, to show the type I cortical granules. **(J)** Dorsal view, to show the type II cortical granules. **(K**,**L)** To show folded part of cell (arrows). Scale bars = 50 μm **(A–C)**, 15 μm **(E–H)**.

**FIGURE 6:**
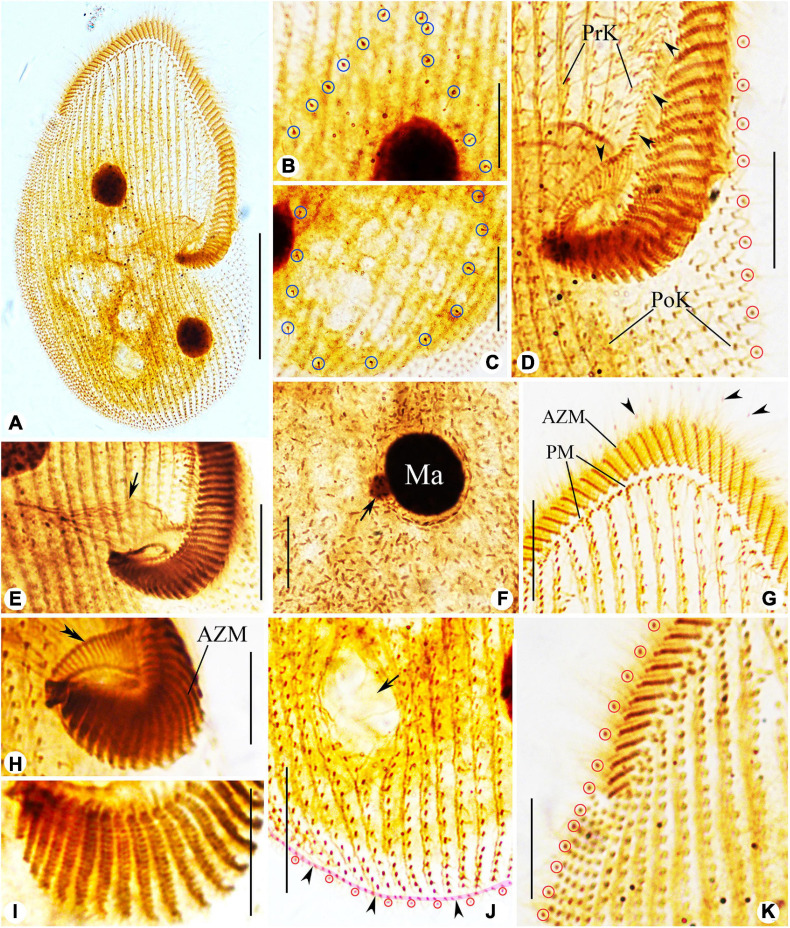
Photomicrographs of *Peritromus kahli* **(E**,**F**,**H**,**I)** population-I and **(A–D**,**G,J,K)** population-II after protargol staining. **(A)** Ventral view of a typical individual. **(B**,**C)** Dorsal views, to show the internal dorsal kinetids (blue circles). **(D**,**E**,**H**,**I)** Detail of oral area; arrowheads indicate the paroral membrane, arrow indicates the cytostome, and double arrowhead marks the fiber bundles, and red circles mark the external dorsal kinetids. **(F)** To show a macronuclear nodule and its associated micronucleus (arrow). **(G)** Ventral view of anterior portion of cell; arrowheads mark the external dorsal kinetids. **(J)** Ventral view of posterior region of cell; arrow indicates a wide gap (possibly the cytoproct) between kineties; pink line (arrowheads) highlights the caudal margin kinety, and red circles mark the external dorsal kinetids. **(K)** Ventral view of right front, and red circles mark the external dorsal kinetids. Abbreviations: AZM, adoral zone of membranelles; PM, paroral membrane; PoK, postoral kineties; PrK, preoral kineties; Ma, macronucleus. Scale bars = 50 μm **(A)**, 15 μm **(B–G**,**K)**, 30 μm **(J)**, and 10 μm **(H**,**I)**.

Body size 100–135 μm × 65–75 μm *in vivo*, 80–145 μm × 60–95 μm after protargol staining (Table 1), length to width ratio approximately 1.5:1 (Figures 4A, 5A). Body outline generally reniform with both ends widely rounded; right cell margin slightly concave, left margin slightly distinctly convex (Figure 5A). Cell strongly dorsoventrally flattened (Figure 5C). With an irregular hump on dorsal side, edge of hump decorated with several wart-like prominences through which spine-like cilia (belonging to internal dorsal kinety) project (Figures 4E, 5F). Adoral zone of membranelles (AZM) commences in anterior quarter near the right margin of the cell, extends around anterior end, continues along the left margin, proximal portion bends toward the cytostome that is located in a narrow depression near the left side in mid-region of the body (Figures 4A,D, 5A,D,E, 6A,D). Pellicle flexible and thin, cell margin often folded. Two types of cortical granules: type I ellipsoidal, dark grayish, about 0.4 μm × 0.2 μm, located between ventral kineties (Figures 4B, 5I); type II spherical, dark greenish, about 0.4 μm in diameter, irregularly located on dorsal side (Figures 4C, 5J). Cytoplasm highly transparent and colorless (Figure 5A). Middle part of the cell opaque and dark gray due to the presence of numerous small globules (about 1–2 μm in diameter) and food vacuoles (about 8–18 μm across, containing small algae) (Figures 4A, 5A). Contractile vacuole absent. Two ellipsoidal macronuclear nodules, on average 17 μm × 13 μm in size, one positioned in anterior right 1/3, the other positioned in posterior left 1/3 of the body (Figures 4E, 5B,G); two micronuclei, each closely associated with a macronuclear nodule (Figure 6F). Locomotion usually by crawling slowly on substrate. When stimulated or disturbed, cells contract and adhere firmly to the substrate.

Infraciliature as shown in Figures 4, 6, including oral apparatus, three kinds of longitudinal ventral kineties, one caudal margin kinety and two circles of dorsal kineties (Table 1). AZM and paroral membrane almost in parallel, terminating proximally near cytostome. AZM composed of about 63–84 membranelles with cilia about 15–20 μm long *in vivo*. Paroral membrane composed of numerous obliquely oriented rows of two or three kinetosomes arranged in a line with all kinetosomes ciliated. Eighteen to 23 fiber bundles, about 2–3 μm long *in vivo*, located between paroral membrane and cytostome (Figures 5E, 6H). Numerous short, rod-like structures, similar to extrusomes, scattered in cytoplasm (Figure 6F). Ventral kineties composed of dikinetids with both kinetosomes ciliated (cilia about 8–10 μm long *in vivo*), including 3–8 short preoral kineties (PrK), 6–17 slightly shortened postoral kineties (PoK), 17–24 bipolar kineties (BK), and a caudal margin kinety surrounding the margin of posterior half of ventral side (Figures 4J, 6J). Preoral kineties located anterior of cytostome, postoral kineties located posterior of AZM. Bipolar kineties almost covering entire ventral surface. Caudal margin kinety located between somatic kineties and external dorsal kineties (Figure 6J). Wide gap near posterior end of ventral surface of cell, probably location of cytoproct (Figures 4J, 6J).

Dorsal kineties comprise one external and one internal dorsal kinety (Figure 4E). External dorsal kinety forming a complete circle around margin between ventral and dorsal sides, cilia about 6 μm long *in vivo* (Figures 4E, 5H). Internal dorsal kinety composed of 17–34 kinetosomes, forming a complete circle around margin of dorsal hump, cilia about 10 μm long *in vivo* (Figures 4E, 5F).

### Morphology of Population-II

Population-II agrees well with population-I in all features (Figures 4F,I,J, 5B,K,L, 6A–D,G,J,K and Table 1), except for (1) the body size *in vivo* (145–175 μm × 80–110 μm vs. 100–135 μm × 65–75 μm); (2) the number of adoral membranelles (71–94 vs. 63–84); (3) the number of postoral kineties (11–22 vs. 6–17); and (4) the number of kinetosomes in internal dorsal kinety (17–34 vs. 17–25).

### SSU rDNA Sequences and Phylogenetic Analyses

**FIGURE 7:**
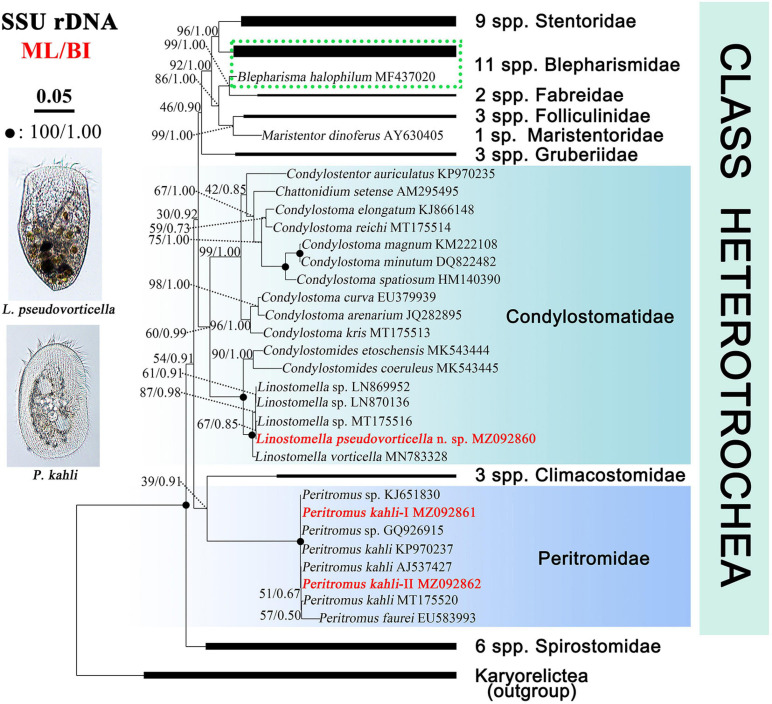
Maximum likelihood (ML) tree inferred from SSU rDNA sequences showing positions of *Linostomella pseudovorticella* n. sp. and *Peritromus kahli.* Newly obtained sequences are in bold red font. Numbers near nodes represent non-parametric values of ML out of 1,000 replicates and Bayesian inference (BI) posterior probabilities. The scale bar corresponds to five substitutions per 100 nucleotide positions.

The SSU rDNA sequences are deposited in the GenBank database. The lengths, GC contents, and accession numbers are as follows: *L. pseudovorticella* n. sp. (1,582 bp, 47.16%, MZ092860), *P. kahli* population-I (1,602 bp, 44.57%, MZ092861), and *P. kahli* population-II (1,602 bp, 44.63%, MZ092862). The SSU rDNA sequences of the two populations of *P. kahli* differ from each other by three nucleotides. The ML and BI analyses based on the SSU rDNA sequences generated phylogenetic trees with nearly identical topologies; therefore, only the ML tree is shown here with support values from both algorithms (Figure 7).

The genus *Linostomella* is monophyletic as all members grouped into one clade with full support (ML/BI, 100%/1.00). It is sister to the genus *Condylostomides* with full support (ML/BI, 100%/1.00) within the family Condylostomatidae. In the *Linostomella* clade, *L. pseudovorticella* n. sp. clusters with *Linostomella* sp. (MT175516) with moderate support (ML/BI, 67%/0.85), forming a subclade that is sister to the other subclade that consists of two unidentified *Linostomella* sequences. These four sequences form a maximally supported group that is sister to *L. vorticella* (MN783328).

The family Peritromidae forms a maximally supported clade that is sister to the family Climacostomidae although with only poor to moderate support (39%/0.91). The internal relationships within the family Peritromidae remain unresolved as indicated by the low support values of the branches. *P. kahli* (MT175520) is sister to *P. faurei* (EU583993) (ML/BI, 57%/0.50), followed by *P. kahli* (AJ537427) and the newly obtained *P. kahli* population-II (MZ092862) (ML/BI, 51%/0.67). This cluster forms a polytomy with two populations of *P. kahli* (KP970237 and population-I MZ092861) and two unclassified *Peritromus* species (KJ651830 and GQ926915).

## Discussion

### Comments on *Linostomella pseudovorticella* n. sp.

The genus *Linostomella* was erected for *L. vorticella*, in 1999, the type species by monotypy. The taxonomy and nomenclature of this special species have long been ambiguous due to its unusual morphological characteristics (Ehrenberg, 1833; Wrześniowski, 1870; Penard, 1922; Jankowski, 1978). Over the past two decades, the phylogenetic position of this species was gradually revealed (Foissner et al., 1992, 1999; Lynn, 2008). Chi et al. (2020) redescribed *L. vorticella* based on both morphological and molecular information and supplied a taxonomic revision of the genus *Linostomella*.

**FIGURE 8:**
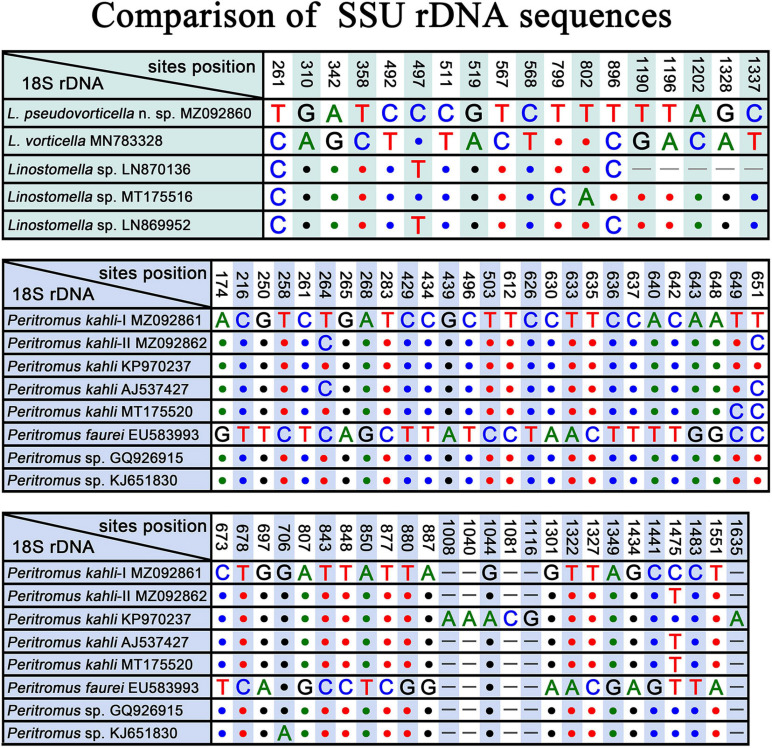
SSU rDNA sequence comparisons showing the unmatched nucleotides between *Linostomella pseudovorticella* n. sp. and related species, and the unmatched nucleotides among *Peritromus kahli*, *Peritromus faurei*, and related species. Nucleotide positions are given at the top of each column. Insertions and deletions are compensated by introducing alignment gaps (–). Matched sites are represented by dots (.).

Three populations of *L. vorticella* have previously been reported with data on the infraciliature, and all three overlap in terms of the number of somatic kineties, i.e., 39–45 in a Rheinland-Pfalz population, 26–45 in a Salzburg population, and 37–51 in a Qingdao population (Packroff and Wilbert, 1991; Foissner et al., 1999; Chi et al., 2020). Among these three populations, the morphological characters of the Qingdao population were described in the most detail, and the molecular phylogeny of this population was also analyzed based on SSU rDNA sequence data. We therefore compare *L. pseudovorticella* n. sp. with the Qingdao population of *L. vorticella* from which it can be separated by: (1) the number of somatic kineties (48–67, mean 56.5 in *L. pseudovorticella* vs. 26–51, mean of 42.4 in *L. vorticella*); (2) the arrangement of the paroral membrane (two parallel rows of kinetosomes in *L. pseudovorticella* vs. two rows of kinetosomes arranged in “zig-zag” pattern in *L. vorticella*); (3) the presence of a distinct glabrous area at the posterior end of the cell in *L. pseudovorticella* (vs. posterior glabrous area lacking in *L. vorticella*); and (4) the SSU rDNA sequence of *L. pseudovorticella* (MZ092860) having a 15-bp difference from that of *L. vorticella* (Figure 8). These differences clearly support the validity of *L. pseudovorticella* as a separate species.

Gelei (1954) reported a species named *Condylostoma vorticella*, which resembles *L. vorticella* in all key characters except the number of somatic kineties (60–70 vs. 26–51 in *L. vorticella*). Although the original description was very brief, we believe that this species is conspecific to the new species because they share the same ciliary pattern. In addition, SSU rDNA sequences of three unidentified *Linostomella* species (LN870136, MT175516, and LN869952) in the GenBank database differ from our new species at three sites (Figure 8), indicating that these three *Linostomella* spp. are probably conspecific with *L. pseudovorticella* n. sp.

### Comments on *Peritromus kahli* Villeneuve-Brachon, 1940

The genus *Peritromus* was established by Stein (1863) with *Peritromus emmae* as the type species. In the following 150 years, another 15 nominal species were described (Fauré-Fremiet, 1924; Kirby, 1934; Bullington, 1940; Villeneuve-Brachon, 1940; Ozaki and Yagiu, 1941; Wailes, 1943; Borror, 1963, 1968; Dragesco, 1965; Tuffrau, 1967; Chardez, 1983; Carey, 1992; Song and Wilbert, 1997; Rosati et al., 2004). Currently, only two species in this genus have been described using modern methods, namely, *P. faurei* and *P. kahli*.

*Peritromus kahli* was first isolated by Villeneuve-Brachon (1940) from French coastal waters of the Mediterranean Sea, and the living morphological characters were described in brief. Although *P. kahli* has been repeatedly reported and reinvestigated in faunal or ecological investigations, important taxonomic information was missing (Borror, 1963; Carey, 1992). Sometimes, *P. kahli* was regarded as a synonym of *P. faurei* (for example, Song and Wilbert, 1997). The first detailed taxonomic information for *P. kahli* based on modern methods, including ultrastructural and SSU rDNA sequence data (AJ537427), was provided by Rosati et al. (2004). In the present work, we provide details of the ciliature pattern and SSU rDNA sequences of two subtropical populations (MZ092861 and MZ092862). Morphologically, *P. kahli* differs from *P. faurei* in the ciliary pattern, i.e., 17–28 bipolar kineties, 6–22 postoral kineties, 63–94 adoral membranelles in *P. kahli* vs. 15–18 bipolar kineties, 5–8 postoral kineties, and 48–51 adoral membranelles in *P. faurei* (Song and Wilbert, 1997). In addition, the SSU rDNA sequences of these two species differ from each other by more than 40 nucleotides, supporting the validity of *P. kahli* and *P. faurei* as separate species.

The four best-documented populations of *P. kahli* exhibit small differences in their morphology, e.g., body shape, number of ventral kineties, and number of adoral membranelles (see Table 2). In addition, the differences in the SSU rDNA sequences among the two present populations and other populations with molecular data range from 0 to 3 nucleotides (except for *P. kahli* KP970237) (Figure 8). All the nucleotide differences between *P. kahli* (KP970237) and other populations are located in the conserved region for the genus *Peritromus*, so it is likely that these differences are due to low quality sequencing. Unfortunately, lack of morphological data for *P. kahli* (KP970237) means that it is not possible to compare it with other populations.

**TABLE 2:** Comparison among different *Peritromus* species.

Population	*P. kahli* population-I present study	*P. kahli* population-II present study	*P. kahli* Villeneuve-Brachon (1940)	*P. kahli* Rosati et al. (2004)	*P. faurei* Song and Wilbert (1997)
Place of sampling	Meishan Island, Ningbo, China	Xiangshan Bay, Ningbo, China	Mediterranean Sea, France	Perros Guirec, Manche, France	Qingdao, China
Body shape and length–width ratio *in vivo*	115 μm × 70 μm, 1.64	145 μm × 95 μm, 1.54	135 μm × 85 μm, 1.60	116 μm × 68 μm, 1.70	60 μm × 45 μm, 1.33
Presence and definition of dorsal ornamentations	Not observed^a^	Not observed^a^	Present, called “cornettes”	Present, called “chalice-like structures”	Not observed
Number of ventral kineties^b^	26–39	34–49	∼40^c^	25–33	20–26
Number of adoral membranelles	63–84	71–94	∼73^c^	60–65	48–51

*^*a*^Lacking ultrastructural study, dorsal ornamentations were not observed in present study.*

*^*b*^The number of ventral kineties is the sum of bipolar kineties and postoral kineties according to Rosati et al. (2004).*

*^*c*^From the drawings in the Villeneuve-Brachon (1940).*

### Phylogenetic Analyses Based on SSU rDNA Sequences

Phylogenetic relationships among families in the class Heterotrichea remain unresolved, as previous studies have concluded that either Peritromidae or Spirostomidae could be the basal group in SSU rDNA trees (Shazib et al., 2014; Fernandes et al., 2016; Yan et al., 2016; Chen et al., 2017; Chi et al., 2021). In the present study, Spirostomidae branches before Peritromidae, which is supported by the morphological data and is consistent with the findings of Fernandes et al. (2016) and Chi et al. (2020). Peritromidae is more morphologically complex than Spirostomidae, as the ciliary pattern on both the ventral and dorsal sides of the former family is conspicuously differentiated, and the paroral membrane is prominent and well-developed. However, some molecular studies did not support the early divergence of Spirostomidae (Rosati et al., 2004; Shazib et al., 2014; Yan et al., 2016; Chen et al., 2017). The grouping of Peritromidae and Climacostomidae is questionable, as the tree topology is far from stable. Furthermore, these two families have relatively few morphological similarities with each other. Therefore, more data from additional species are needed to determine the evolutionary relationships among families of the class Heterotrichea.

Stentoridae, Blepharismidae, Fabreidae, Folliculinidae, Maristentoridae, and Gruberiidae are closely related in our SSU rDNA tree, which is consistent with previous analyses (Shazib et al., 2014, 2016, 2019; Fernandes et al., 2016; Chen et al., 2017, 2018; Luo et al., 2019; Chi et al., 2020, 2021). The monophyletic family Condylostomatidae is divided into two subclades, *Condylostomides* + *Linostomella* (freshwater habitat) and *Chattonidium* + *Condylostentor* + *Condylostoma* (marine water habitat). The genus *Linostomella* is monophyletic and is closely related with *Condylostomides*, which is consistent with previous studies (Rossi et al., 2016; Chi et al., 2020, 2021). The findings of the present study support the assertion that habitat preference is a phylogenetically informative character among these taxa (Chi et al., 2021). However, the genus *Condylostoma* failed to form a monophyletic group in many phylogenetic analyses (Miao et al., 2009; Shazib et al., 2014; Yan et al., 2015; Fernandes et al., 2016; Chen et al., 2020; Chi et al., 2021). The result in the present study is the same as before and supports the view in Chen et al. (2020) that *Condylostoma* is likely a paraphyletic group.

## Data Availability Statement

The datasets presented in this study can be found in online repositories. The names of the repository/repositories and accession number(s) can be found in the article/Supplementary Material.

## Author Contributions

DJ performed the experiments and drafted the manuscript. JH performed the phylogenetic section. TY and XZ helped to collect the samples. AW and SA-F checked all the taxonomic works and helped to writing the manuscript. XC supervised and coordinated the work. All authors read and approved the final manuscript.

## Conflict of Interest

The authors declare that the research was conducted in the absence of any commercial or financial relationships that could be construed as a potential conflict of interest.

## Publisher’s Note

All claims expressed in this article are solely those of the authors and do not necessarily represent those of their affiliated organizations, or those of the publisher, the editors and the reviewers. Any product that may be evaluated in this article, or claim that may be made by its manufacturer, is not guaranteed or endorsed by the publisher.

## Footnotes

1
http://guidance.tau.ac.il/

2
http://molevol.cmima.csic.es/castresana/Gblocks_server.html

3
https://www.phylo.org
