# Supplementary material for: Taxonomy and SSU rDNA-Based Phylogeny of Two Heterotrich Ciliates (Ciliophora, Heterotrichea) Collected From Subtropical Wetlands of China, Including the Description of a New Species, Linostomella pseudovorticella n. sp
Source: Front Microbiol. 2021 Sep 7;12:719360. doi: 10.3389/fmicb.2021.719360 (PMC8453171; doi:10.3389/fmicb.2021.719360)
Supplement: Supplementary file 1 [file Table_1.DOCX]

**TABLE S1 |** GenBank accession numbers of all taxa in **FIGURE 7** except for the members of outgroup (new sequences are in bold).

| Species | GB number | Species | GB number |
| --- | --- | --- | --- |
| Family Maristentoridae |  | Family Stentoridae |  |
| *Maristentor dinoferus* | AY630405 | *Stentor coeruleus* | KP970244 |
|  |  | *Stentor igneus* | KP970245 |
| Family Fabreidae |  | *Stentor roeseli* | KJ651826 |
| *Fabrea salina* | KM222110 | *Stentor muelleri* | KJ651824 |
| *Fabrea salina* | EU583991 | *Stentor polymorphus* | JQ282898 |
|  |  | *Stentor tartari* | KY979516 |
| Family Folliculinidae |  | *Stentor elegans* | FN659817 |
| *Ampullofolliculina lageniformis* | MK775465 | *Stentor multiformis* | FN659822 |
| *Folliculina simplex* | EU583992 | *Stentor amethystinus* | KP970242 |
| *Eufolliculina uhligi* | U47620 |  |  |
|  |  | Family Condylostomatidae |  |
| Family Gruberiidae |  | *Chattonidium setense* | AM295495 |
| *Gruberia foissneri* | MN783327 | *Condylostentor auriculatus* | KP970235 |
| *Gruberia* sp*.* | L31517 | *Condylostoma kris* | MT175513 |
| *Gruberia lanceolata* | MH024390 | *Condylostoma curva* | EU379939 |
|  |  | *Condylostoma arenarium* | JQ282895 |
| Family Climacostomidae |  | *Condylostoma reichi* | MT175514 |
| *Climacostomum virens* | KJ651814 | *Condylostoma elongatum* | KJ866148 |
| *Climacostomum virens* | EU583990 | *Condylostoma spatiosum* | HM140390 |
| *Climacostomum virens* | KP970234 | *Condylostoma magnum* | KM222108 |
|  |  | *Condylostoma minutum* | DQ822482 |
| Family Peritromidae |  | *Condylostomides etoschensis* | MK543444 |
| *Peritromus kahli* | MT175520 | *Condylostomides coeruleus* | MK543445 |
| *Peritromus faurei* | EU583993 | *Linostomella vorticella* | MN783328 |
| *Peritromus kahli* | AJ537427 | *Linostomella* sp. | LN870136 |
| ***Peritromus kahli* population-I** | MZ092861 | *Linostomella* sp. | MT175516 |
| *Peritromus* sp. | KJ651830 | *Linostomella* sp. | LN869952 |
| *Peritromus kahli* | KP970237 | ***Linostomella pseudovorticella* n. sp.** | MZ092860 |
| ***Peritromus kahli* population-II** | MZ092862 |  |  |
| *Peritromus* sp. | GQ926915 | Family Spirostomidae |  |
|  |  | *Spirostomum ambiguum* | KJ651819 |
| Family Blepharismidae |  | *Spirostomum subtilis* | MK929558 |
| *Blepharisma sinuosum* | KP970229 | *Spirostomum semivirescens* | MH295830 |
| *Blepharisma undulans* | KR815912 | *Spirostomum minus* | MK929559 |
| *Blepharisma americanum* | KP970223 | *Spirostomum yagiui* | KU848227 |
| *Blepharisma japonicum* | KP970228 | *Spirostomum dharwarensis* | MN160212 |
| *Blepharisma musculus* | KJ651813 | *Spirostomum caudatum* | KJ651820 |
| *Blepharisma steini* | KP970224 | *Spirostomum teres* | MK929560 |
| *Blepharisma penardi* | KR815913 | *Pseudoblepharisma tenue* | MK543441 |
| *Blepharisma hyalinum* | KP970225 | *Anigsteinia clarissima* | KR815914 |
| *Blepharisma elongatum* | AM713186 | *Anigsteinia clarissima* | KM222109 |
| *Blepharisma bimicronucleatum* | KX119522 | *Anigsteinia* sp. | KU848242 |
| *Blepharisma halophilum* | MF437020 |  |  |
